# Supplementary material for: The Cumulative Formation of R-loop Interacts with Histone Modifications to Shape Cell Reprogramming
Source: Int J Mol Sci. 2022 Jan 29;23(3):1567. doi: 10.3390/ijms23031567 (PMC8835745; doi:10.3390/ijms23031567)
Supplement: Supplementary file 1 [file ijms-23-01567-s001.zip › ijms-15558986-Supplementary Figures.pdf]

International Journal of
Molecular Sciences

MDPI

Supplements

The Cumulative Formation of R-loop Interacts with Histone Modifications to Shape Cell Reprogramming

Hanshuang Li [1,†], Chunshen Long [1,†], Yan Hong [1], Lemuge Chao [1], Yong Peng [2] and Yongchun Zuo [1,*]

Supplementary Figures

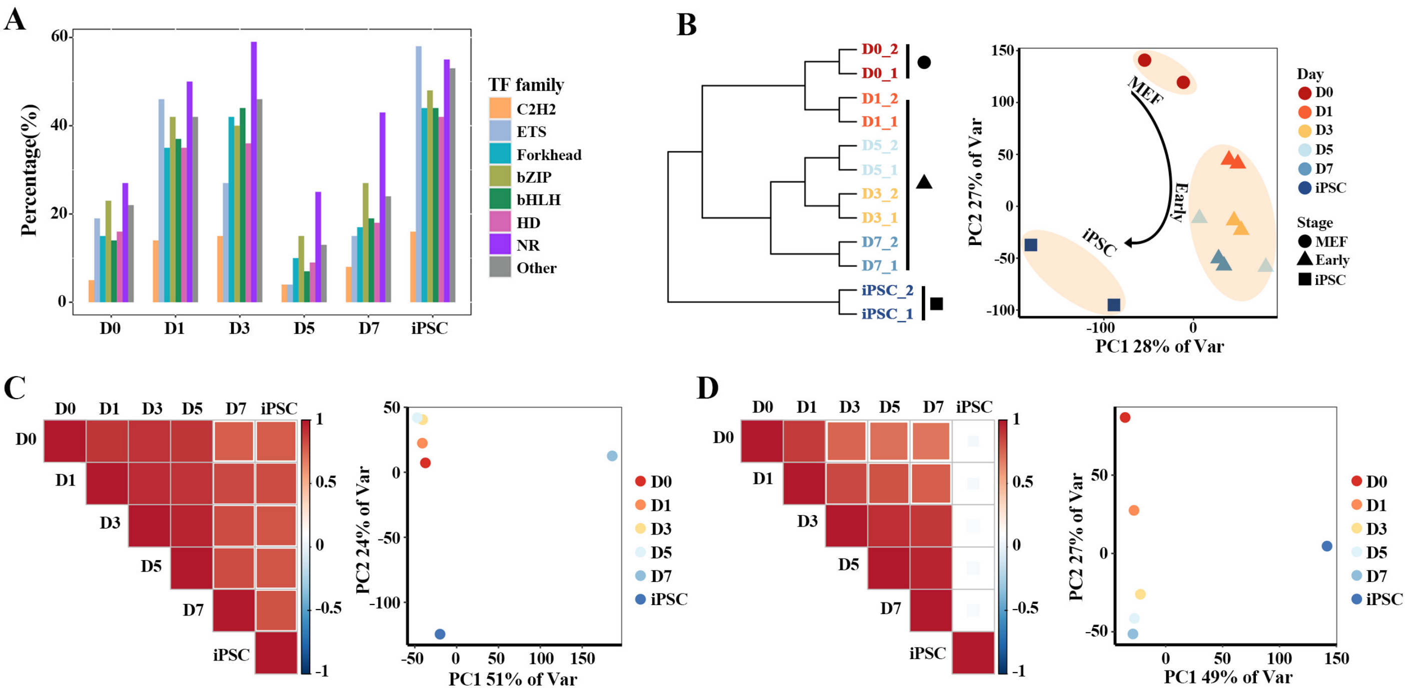

Figure S1. Distribution of R-loops on different elements and analysis of histone modification patterns. **(A)** The distribution of R-loop peaks on different TF family. **(B)** Hierarchical clustering (left) and Principal component analysis (PCA, right) of global transcriptome at different reprogramming stages. Black arrow represents the trajectory of MEF reprogramming to the iPSC state. **(C)** Correlation analysis (left) and PCA (right) of global H3K4me3 binding signals at different reprogramming stages. **(D)** Correlation analysis (left) and PCA (right) of global H3K27me3 binding signals at different reprogramming stages.

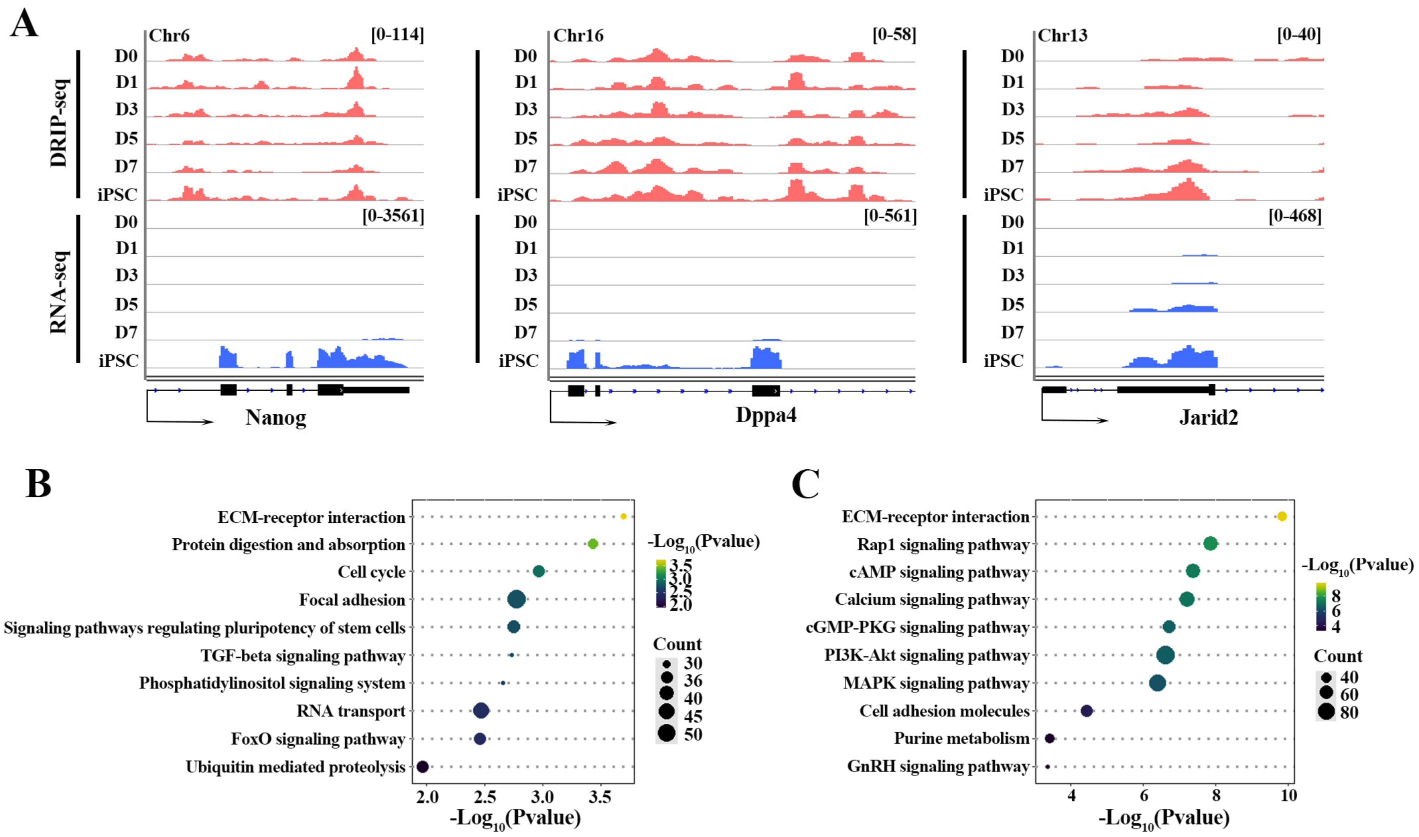

Figure S2. KEGG pathway enrichment analysis of co-differential genes. **(A)** Representative genome browser snapshot of R-loop signals and genes expression levels during reprogramming progress. **(B)** The top 10 significant KEGG pathways (P value < 0.01) for up-regulated genes with up-regulated R-loop signals (n = 3689, as shown in Figure 2E) between adjacent reprogramming stages. **(C)** The top 10 significant KEGG pathways (P value < 0.01) for down-regulated genes with down-regulated R-loop signals (n = 3290, as shown in Figure 2F) between adjacent reprogramming stages.

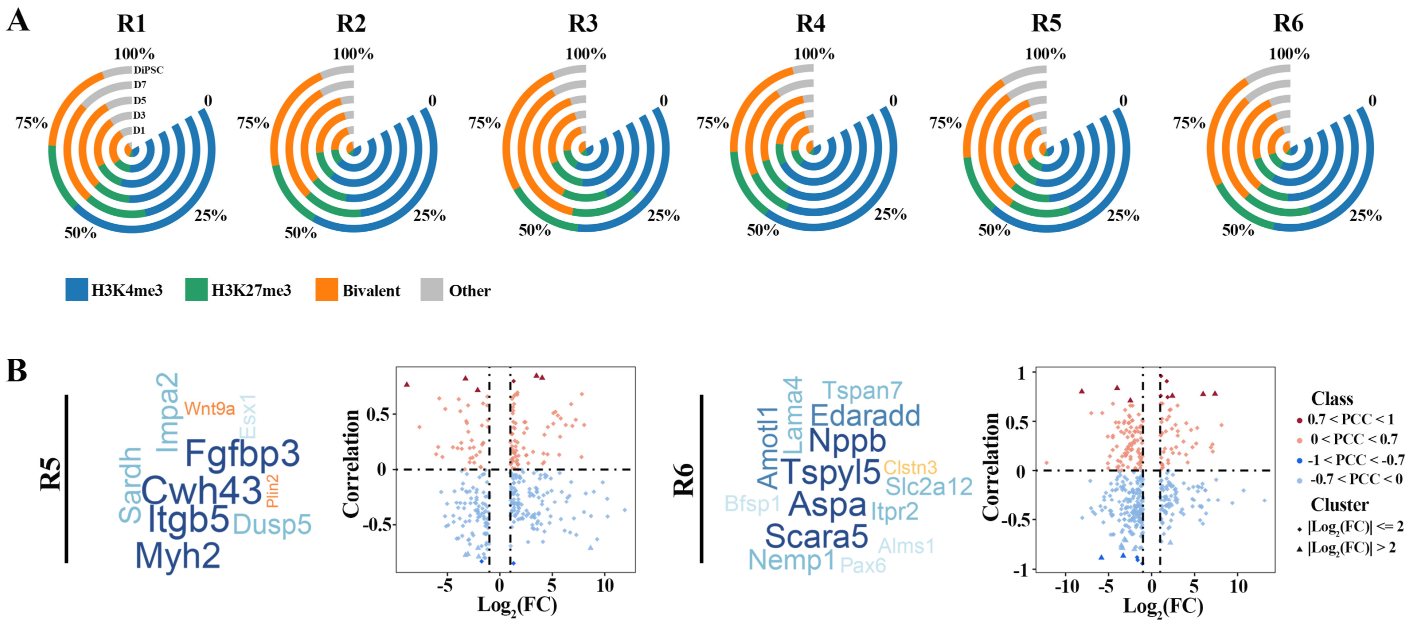

Figure S3. Different histone modification patterns on six clusters. **(A)** Proportion of co-differential genes bound by H3K4me3 (blue), H3K4me3 (green), Bivalent (orange) and other modification in each cluster (R1-R6). **(B)** Integrated analysis of DEGs and differential R-loop signals in R5 and R6. Wordclouds represent the key genes (0.7 < |PCC| < 1; |Log2(FC)| > 2) in each group, both size and color of the word being proportional to the membership value of each gene. Y-axis of the scatter plot indicates the correlation between expression levels and R-loop signals and X-axis indicates logarithmic transformation of the fold change (FC) of the expression level (iPSC vs. D0).
